# Epigenetic IVD Tests for Personalized Precision Medicine in Cancer

**DOI:** 10.3389/fgene.2019.00621

**Published:** 2019-06-28

**Authors:** Jesús Beltrán-García, Rebeca Osca-Verdegal, Salvador Mena-Mollá, José Luis García-Giménez

**Affiliations:** ^1^Center for Biomedical Network Research on Rare Diseases (CIBERER), Institute of Health Carlos III, Valencia, Spain; ^2^INCLIVA Biomedical Research Institute, Valencia, Spain; ^3^Department of Physiology, School of Medicine and Dentistry, Universitat de València (UV), Valencia, Spain; ^4^EpiDisease S.L. Spin-Off of CIBERER (ISCIII), Valencia, Spain

**Keywords:** precision medicine, epigenetic biomarker, In Vitro Diagnostic (IVD), DNA methylation, miRNA, cfDNA, circulating nucleosomes

## Abstract

Epigenetic alterations play a key role in the initiation and progression of cancer. Therefore, it is possible to use epigenetic marks as biomarkers for predictive and precision medicine in cancer. Precision medicine is poised to impact clinical practice, patients, and healthcare systems. The objective of this review is to provide an overview of the epigenetic testing landscape in cancer by examining commercially available epigenetic-based *in vitro* diagnostic tests for colon, breast, cervical, glioblastoma, lung cancers, and for cancers of unknown origin. We compile current commercial epigenetic tests based on epigenetic biomarkers (i.e., DNA methylation, miRNAs, and histones) that can actually be implemented into clinical practice.

## Introduction

Epigenetics, a breakthrough discipline in biomedicine, aims to improve precision medicine by discovering new epigenetic mechanisms and providing new epigenetic biomarkers, therapeutic targets, and epigenetic drugs with potential uses in clinical practice.

Most human diseases have complex multifactorial pathologies that result from a pathogenic polymorphism in human genes, besides epigenetic mechanisms, which can modulate the expression of functional genes. Currently, several IVD molecular-based tests contribute to the development of precision oncology, which already offers viable alternatives for cancer diagnostics and prognostics. The Food and Drug Administration (FDA) lists several IVD tests that have been cleared and approved for diagnostics, which can be consulted by searching *Nucleic Acid-Based Test* (Food and Drug Administration, 2019a) and *List of Cleared or Approved Companion Diagnostic Devices (In Vitro and Imaging Tools)* (Food and Drug Administration, 2019b).

For a given phenotype, there is a causal contribution of genetic mutations, copy number variations, epigenetic control, and altered transcription programs and altered complex metabolic inputs. The contribution of the aforementioned factors renders the use of different approaches necessary to understand the physiopathology of complex and multifactorial diseases. In line with this, epigenetic biomarkers can help early diagnosis, disease progression monitoring, disease outcome prediction, selection and stratification of patients by risk, prediction of future comorbidities, and even the evaluation of the positive or negative effects of therapeutic interventions in specific patient subsets. Among others, DNA methylation and microRNAs are markedly more stable than RNA and proteins, which renders the use of these biomarkers more practical and viable in clinical settings (Faruq and Vecchione, 2015; Hashimoto et al., 2016; García-Giménez et al., 2017b). In particular, DNA methylation, microRNAs, and post-translational modifications of histones offer high stability in biofluids and in samples with a compromised quality, such as formalin-fixed paraffin embedded (FFPE). Other advantages of epigenetic biomarkers over genetic or protein-based biomarkers are as follows: 1) their dynamic nature; 2) they provide information about the gene function; 3) they inform about the specific genetic programs that alter during disease; and 4) most techniques to analyze epigenetic biomarkers (i.e., RT-qPCR) have already been introduced into clinical laboratories. Therefore, epigenetics has a tremendous potential to improve predictive and precision medicine.

Precision Medicine was defined by the National Research Council’s Toward Precision Medicine in 2008 as: “The tailoring of medical treatment to the individual characteristics of each patient … to classify individuals into subpopulations that differ in their susceptibility to a particular disease or their response to a specific treatment. Preventative or therapeutic interventions can then be concentrated on those who will benefit, sparing expense and side effects for those who will not” (Ginsburg and Phillips, 2018). Therefore, precision medicine has started to use potential epigenetic biomarkers in clinical settings.

We recently defined an epigenetic biomarker as “any epigenetic mark or altered epigenetic mechanism which generally serves to evaluate health or disease status and is particularly stable and reproducible during sample processing.” An ideal biomarker can be measured in body fluids (i.e., plasma, serum, saliva, semen, urine, etc.) or primary tissue samples (fresh tissue, cells, single cell isolated, fine-needle aspirates, FFPE, etc.). However, for clinical settings, minimal invasive procedures are preferable. In line with this, human plasma as a source of miRNAs and circulating cell-free DNA (cfDNA) is, therefore, the best option. An ideal epigenetic biomarker for precision medicine applications may cover at least one of the following properties: i) predicts the risk of future disease development (risk); ii) defines a disease (detection); iii) reveals information about the natural history of the disease; iv) predicts the outcome of disease (prognostic); v) responds to therapy (predictive); vi) monitors responses to therapy or medication (therapy monitoring); vii) allows to simultaneously make a diagnosis and perform targeted therapy (theragnosis) (García-Giménez et al., 2017b).

To achieve the precision medicine goals, the current challenge is knowing how to obtain a reliable useful biomarker for clinical routine because, for this purpose, the new biomarker requires high accuracy and robustness (Li et al., 2010; Diamandis, 2012) and cost-effectiveness. It is noteworthy that less than 1% of the biomarkers obtained in biomedical research is finally implemented into the clinical laboratory (Kern, 2012), with an even lower percentage for epigenetic biomarkers. This low percentage of commercialized IVD tests based on epigenetic biomarkers suggests that the precision medicine ecosystem formed by distinct stakeholders (i.e., patients, providers, payers, and regulators) may increase their knowledge about the impact of epigenetic biomarkers on precision medicine, and might also work together to successfully implement this breakthrough technology in clinical practice.

A number of precision medicine applications are contributing to health care improvements by allowing the precise diagnosis of diseases or by identifying specific disease subsets or stages, and by also improving personalized treatments. Specifically, for cancer, which remains the second leading cause of death worldwide, early detection, the identification of cancer subtypes, and the selection of appropriate therapies are crucial to increase the survival of cancer patients. However, the identification of new tumor biomarkers, especially those based on epigenetic biomarkers with the capability to identify tumor origin or cancer subsets, advances in assay technologies, and the development of sophisticated analytical software techniques (i.e., machine learning and artificial intelligence), will help to improve precision medicine in cancer (Ahlquist, 2018).

## Technologies for Epigenetic Biomarker Analyses in Clinical Laboratories

Given the prevalence of the DNA methylation alterations at specific genes under a variety of human disease conditions, a promising future is coming for the DNA methylation analysis as an epigenetic biomarker. In fact, DNA methylation is the best-studied epigenetic modification since it was discovered. In addition, miRNAs have attracted a great deal of interest in clinical research for their role in gene regulation, tissue signaling and cellular homeostasis, their high stability in practically all types of biospecimens, and the relatively easy way by which to measure miRNAs in a wide array of biospecimens. Histone variants and histone post-translational modifications are other potential markers that can be analyzed in a wide array of biospecimens for clinical settings.

Therefore, it is not surprising that most current commercial *in vitro* diagnostic tests are based on either the analysis of DNA methylation of specific genes or the measurement of the relative expression of microRNAs, which can be easily measured by RT-qPCR-based methods (i.e., methyLight, methyl-specific PCR, and methylation-sensitive high-resolution melting) and pyrosequencing technologies (García-Giménez et al., 2017a).

There are other assays based on high-throughput analyses to simultaneously measure several CpG sites. This is, for example, the case of the EPICUP^®^ assay, which is based on using human methylation array Beadchip 450K (Illumina). In the following section, we provide details of a selection of current IVD tests based on epigenetic biomarkers that are currently being commercialized for *in vitro* diagnostic in cancer (**Table 1**).

**Table 1:** Commercially available Epigenetic IVD tests with the potential of improving precision medicine in cancer.

Diseases	Epigenetic biomarkers	Commercial tests	Technology for the analysis	Biospecimen	Sn (%)	Sp (%)
Colorectal cancer	DNA methylation (*NDRG4* and *BMP3*)	*Cologuard* ^®^ *stool-DNA-based test*	Stool-based CRC test	Stool	92.3	86.6
	DNA methylation (*SEPT9*)	*Epi proColon* ^®^ *2.0 test*	MethyLight	CfDNA from blood	75-81	96-91
	DNA methylation (*SDC2*)	*EarlyTect* ^®^ CRC *assay*	MethyLight	CfDNA from blood	87	95.2
	miR-31-3p	*miRPreDX-31-3p*	RT-qPCR	FFPE	NA	NA
Breast cancer	DNA methylation (*PITX2*)	*Therascreen PITX2 RGQ PCR kit.*	MethyLight	FFPE; DNA from blood	NA	NA
Cervical cancer	DNA methylation (*ZNF582*)	*Cervi-M* ^®^ *assay*	Methyl-specific PCR	Epithelial cells from cervical brush	73	80
Glioblastoma	DNA methylation (*MGMT*)	*Therascreen MGMT Pyro Kit*	Pyrosequencing	FFPE, DNA from blood	95-97	NA
Lung cancer	DNA methylation (*SHOX2* and *PTGER4*)	*Epi proLung BL Reflex Assay* ^®^	Methyl-specific PCR	CfDNA from blood	78	96
Cancers of unknown origin	Analysis of 450K CpGs	*EPICUP* ^™^	Human methylation Beadchip 450 K (Illumina)	FFPE	97.7	99.6

cfDNA, circulating cell-free DNA; FFPE, formalin-fixed, paraffin-embedded; Sn, Sensitivity; Sp, specificity; NA, data not available.

## *In Vitro* Diagnostic Tests Based on Epigenetic Biomarkers

### Epigenetic-Based IVD Test for Colorectal Cancer

Colorectal cancer (CRC) (MIM 11 4500) is the third most frequent cancer in men and the second most frequent cancer in women worldwide, and accounts for nearly 10% of cancers (Ferlay et al., 2015). CRC is the second leading cause of death by cancer. Five-year survival rates range from more than 90% for stage I to less than 10% for stage IV CRC (Siegel et al., 2012). CRC is characterized by slow progression from detectable precancerous lesions and has a good prognosis when patients are diagnosed in early stages. Non-invasive fecal immunochemical test (FIT) for hemoglobin detection in stools is the most widely used test, but its sensitivity is relatively low in detecting early stage I CRC (53%) and advanced adenomas (≥ 1.0 cm) (27%) (Morikawa et al., 2005). Therefore, the potential for reducing the burden of CRC by early detection is significant, and efforts are currently being made to develop CRC screening tests and to improve the adherence rates of participation for screening because people scarcely comply with currently available methods (Issa and Noureddine, 2017). The selection of appropriate therapies for CRC patients is also a clinical need. Among the therapies proposed for CRC, anti-epidermal growth factor receptor (EGFR) mAb therapy is not indicated for carriers of RAS mutations [approximately 50% of patients with metastatic CRC because the mutations in the *RAS* gene (mainly in exons 2, 3, and 4 of *KRAS* and *NRAS*) make metastatic CRC patients non responders to anti-EGFRs mAB treatment] (Boleij et al., 2016). So, the identification of additional biomarkers to allow clinicians to select those patients who could benefit by the established therapies is needed.

#### The Cologuard^®^ Stool DNA-Based Test

The first FDA-approved DNA methylation assay for general CRC screening for average-risk adults older than 50 years was Cologuard^®^ (Exact Sciences Corp., Madison, WI). The Cologuard^®^ IVD test is a multitarget stool deoxyribonucleic acid (MT-sDNA) screening test based on the analysis of the methylation levels of genes N-Myc downstream-regulated gene 4 (*NDRG4*) and bone morphogenetic protein 3 (*BMP3*), a mutation in the *KRAS* gene (exon 2, codons 12, 13, using ß-actin as the reference gene), and a non-DNA immunochemical assay for human hemoglobin that allows the precise detection of colon neoplasia (Imperiale et al., 2014). The methylation analysis of *NDRG4* and *BMP3* using *ACTB* (ß-actin) as the reference gene is performed according to the method described by Zou et al. (2012), while fecal hemoglobin biomarker values are obtained by the analytical method described by Lidgard et al. (2013). Cologuard^®^ uses a composite score algorithm that is incorporated into the multitarget stool DNA analytic device software as described by Imperiale et al. (2014).

Cologuard^®^ sensitivity and specificity for CRC detection in a study performed with 9,989 subjects was 92.3% and 86.6%, respectively (Imperiale et al., 2014). Although the Cologuard^®^ test sensitivity was higher than FIT (which measures the presence of blood in the colon in fewer fecal samples) for detecting CRC (92% vs. 74%, p = 0.015), specificity was lower than that shown by the FIT ((87% vs. 95%) (Imperiale et al., 2014). Moreover, the Cologuard^®^ test detected less than half largely advanced adenomas (precancerous lesions), but performs better than the FIT. In fact, the sensitivity for detecting advanced precancerous lesions was 42.4% with DNA testing and 23.8% with the FIT (P < 0.001). These results reinforce the potential of the Cologuard^®^ test as an alternative for surveillance colonoscopy (van Lanschot et al., 2017). However, its high cost and difficult sample pretreatment and management for each analysis type are considered disadvantages for its rapid implementation into clinical routine. Accordingly, the results obtained with the Cologuard^®^ test are delivered to the healthcare provider within 2 weeks from receiving the stool sample.

Despite these disadvantages, both the US Food and Drug Administration and the US Preventive Services Task Force (USPSTF) include the Cologuard^®^ test in their screening exam recommendations (Lin et al., 2016).

#### The Epi proColon^®^ 2.0 Test

The Epi proColon^®^ test (Epigenomics AG, Berlin, Germany) was designed to minimize invasive tests and to increase the adherence rates of the participation of those people screened for CRC. The Epi proColon^®^ test uses peripheral blood samples to analyze the methylation status of the *SEPT9* gene. Septins are essential proteins during cell division, and *SEPT9* hypermethylation has been proposed as a key factor in CRC (Song and Li, 2015). The original assay was designed to extract DNA from 5 ml of plasma samples, bisulfite conversion of DNA, and its purification by a particle-based bis-DNA purification method to improve the recovery of bisulfite-treated DNA, the quantification of converted DNA by real-time PCR, and the subsequent measurement of *SEPT9* methylation, and *ACTB* (ß-actin) as a reference gene, by real-time PCR in a Lightcycler LC480 system (Roche Applied Science) and the Quantitect Multiplex PCR mastermix (Qiagen) (DeVos et al., 2009). Epi proColon^®^ 2.0 (Epigenomics Inc., Germany) was approved by the FDA in 2016 as the first blood test intended for early CRC detection. In a large clinical trial using 1,544 plasma samples from the PRESEPT study cohort (ClinicalTrials.gov, Trial Registration ID: NCT00855348), Epi proColon^®^ demonstrated high sensitivity, which ranged from 77.0% to 81.4%, and specificity from 77.9% to 92.1% (Potter et al., 2014). However, some studies have shown some flaws in the use of Epi proColon^®^ to diagnose CRC, such as its lower sensitivity for stage I than for stages II, III, or IV (Jin et al., 2015). A large multicenter prospective study using blood samples from 53 CRC cases and from 1,457 subjects without CRC from the PRESEPT cohort (ClinicalTrials.gov, Trial Registration ID: NCT00855348) showed low sensitivity (48.2%) for detecting CRC and very low sensitivity (11.2%) for identifying advanced adenoma, with 91.5% specificity (Church et al., 2014). One noteworthy result was that the positive detection rate of the *SEPT9* methylation assay increased exponentially as colorectal lesions became more severe and with more advanced CRC stages (Song et al., 2018), although a negative result does not guarantee absence of cancer.

The results obtained by Song et al. (2018) and He et al. (2018) suggest that the methylation status of *SEPT9* could be applied to CRC stage, size, invasion depth, future risk assessment, metastasis, disease progression monitoring, and therapeutic effect evaluation. A possible flaw of this test is that Epi proColon^®^ detected the methylated status of the same region of the *SEPT9* gene in some patients affected by other cancers (i.e., prostate, breast, lung or other diseases, hypertension, hyperlipidemia, diverticulitis, chronic gastritis, or cardiovascular) and according to their age (Ørntoft et al., 2015). Indeed, the Epi proColon^®^ test was positive in 72 (42%) of 173 patients with other cancers and positive in 33 of 191 patients (17%) with other diseases. In addition, an active clinical trial was run to evaluate the potential of the Epi proColon^®^ test for detecting hepatocellular carcinoma among cirrhotic patients (ClinicalTrials.gov, Trial Registration ID: NCT03311152). These scenarios suggest the potential of this test to diagnose other cancers, such as breast cancer, as demonstrated by Shen et al. (2018), but also the inconvenience of the positive results given for cancer patients who were negative for CRC.

It is worth mentioning that colonoscopy remains the universal gold standard method for CRC diagnostics. In Europe and Asia Pacific, only the use of fecal occult blood test (gFOBT) or quantitative FIT for non-invasive screening is still recommended. However, Chinese guidelines have recently recommended using the test as a complement to other diagnostic approaches, like the guaiac-based gFOBT. In the United States, Epi proColon^®^ is not intended to replace the CRC screening tests recommended by clinical guidelines (i.e., colonoscopy, sigmoidoscopy, and gFOBT), but the Epi proColon^®^ test was FDA-approved for CRC screening those patients unwilling or unable to be screened by recommended methods following guidelines.

#### The EarlyTect^®^ Colorectal Cancer Assay

The EarlyTect^®^ CRC test (Genomictree Inc. Daejeon, South Korea) has recently received CE-IVD certification for the diagnosis of CRC. The EarlyTect^™^-GI Syndecan2 Methylation Assay is an IVD assay that uses cfDNA isolated from 0.5 ml of serum to analyze the methylation status of *SDC2* (Syndecan-2).

Previous studies have demonstrated the potential of the analysis of the methylation status of the *SDC2* gene for the early diagnosis of CRC. For example, the studies performed by Mitchell et al. (2016) showed lower sensitivity (59%), but relatively good specificity (84%), of methylation-specific PCR assays (probe-based MethyLight assays) for *SDC2* in the early detection of CRC. At this point, it is worth mentioning that the amplicon selected to study the methylation status of this gene slightly differed (420 bp downstream to the CpG proposed by Oh et al., 2017).

More recent studies performed by Oh et al. (2017), which evaluated the methylation analysis of *SDC2* to detect CRC using isolated DNA from stool samples, demonstrated a good sensitivity of 90.0% for detecting CRC and 33.3% for small polyps, with a specificity of 90.9%. Furthermore, these authors demonstrated that the *SDC2* methylation level was linked to cancer severity in CRC patients in stages I to IV (n = 50). Similarly, Niu et al. (2017) evaluated the methylation levels of the *SDC2* gene in 497 stool samples and found sensitivities of 81.1% and 58.2% for detecting CRC (n = 196) and adenoma (≥1 cm) (n = 122), respectively, with 93.3% specificity. These results were comparable to that observed by Park et al. (2018), who found that the *SDC2* gene methylation analysis performed with methyl-specific PCR in bowel lavage fluid collected during colonoscopy could detect CRC and precancerous lesions. In this study, *SDC2* methylation was positive in 100% of villous adenoma, high-grade dysplasia, and hyperplastic polyp biopsies in 88.9% of tubular adenoma samples and in 0% of normal mucosal samples. These findings indicate the potential of *SDC2* methylation as a biomarker for early CRC detection with a sensitivity of 80% and a specificity of 88.9%.

The clinical validation of *SDC2* methylation in serum DNA from the CRC patients (n = 131) in stages I to IV (stage I, 26; II, 57; III, 36; IV, 12) and from healthy individuals (n = 125) by quantitative methylation-specific PCR using the methylation-specific TaqMan probe demonstrated 87% sensitivity [114/141; 95% confidence interval (CI), 80.0% to 92.3%] and 95.2% specificity (10/125; 95%CI, 89.8% to 98.2%) (Oh et al., 2013). The sensitivity of the patients in stage I was particularly high with 92%, which suggests the potential utility of this test for early CRC detection and identification of precancerous lesions, such as polyps.

A recent observational clinical trial conducted with the EarlyTect^®^ CRC test (ClinicalTrials.gov, Trial Registration ID: NCT03146520) was designed to validate the clinical performance of the *EarlyTect* ^®^ *Colon Cancer test* in stool DNA to detect CRC in a case-control study with 634 participants (Dae Han et al., 2019). Of the 585 evaluated subjects, 245 had CRC, 44 had various sized adenomatous polyps, and 245 obtained negative colonoscopy results. The EarlyTect^®^ CRC test gave an overall sensitivity of 90.2% with area under the curve (AUC) of 0.902 in detecting CRC (0–IV) not associated with tumor stage, and a specificity of 90.2%. The sensitivity for detecting early stages (0-II) was 89.1% (114/128). The EarlyTect^®^ CRC test also detected 66.7% (2/3) and 24.4% (10/41) of advanced and non-advanced adenomas, respectively (Dae Han et al., 2019).

Genomictree Inc. has performed experiments to evaluate the cross-reactivity of the EarlyTect^®^ CRC test in an interim clinical validation with stool DNA from 50 CRC patients (stage I, 10; II, 16; III, 14; IV, 10), 14 irritable bowel syndrome (no colonoscopy was performed), 4 with acute colitis, 11 Crohn’s disease (colonoscopy was performed), 14 ulcerative enteritis (colonoscopy was performed), and 50 healthy subjects (endoscopy was not performed). In this study, the sensitivity was 90.0% (45/50) and specificity was 90.9% (5/55). The methylation positivity for *SDC2* was observed in 14.3% (2/14) of the irritable bowel syndrome patients, 25.0% (1/4) of the acute colitis patients, and 35.7% (5/14) of the ulcerative colitis patients, while no Crohn’s disease case was positive for the EarlyTect^®^ assay. Notably, sensitivity was 84.6% (22/26) in CRC in stages I and II, which suggests the potential applicability of this test for colorectal detection testing using stool DNA.

#### miRPreDX-31-3p

The miRpredX-31-3p kit (IntegraGen S.A., France) is a CE-IVD marked theranostic test intended to identify patients with metastatic CRC who can benefit from anti-EGFR (epidermal growth factor receptor) therapy. The miRpredX-31-3p kit quantifies relative miR-31-3p levels by RT-qPCR from the total RNA extracted from FFPE samples in primary tumors of patients with metastatic CRC, using a cutoff value of 1.36 for the miR-31-3p expression level to define patients as being low or high expressers of this miRNA (Ramon et al., 2018).

miRpredX-31-3p predicts the potential clinical benefits associated with first-line anti-EGFR (epidermal growth factor receptor) therapy compared with anti-vascular endothelial growth factor receptor (VEGF) therapy or when second or further lines of treatment with anti-EGFR mAB therapy is more beneficial *versus* chemotherapy alone for multiple patient outcomes (Laurent-Puig et al., 2015). Specifically, on one hand, a low miR-31-3p expression in affected tissue is associated with a 12-month survival advantage and a 40% reduced risk of death when using anti-EGFR (cetuximab) therapy *versus* anti-VEGF (bevacizumab) therapy in patients with metastatic CRC. On the other hand, those patients expressing high miR-31-3p levels displayed no differences in outcomes when treated with either anti-EGFR or anti-VEGF therapy (Laurent-Puig et al., 2015; Laurent-Puig et al., 2017). Furthermore, the miR-31-3p expression was evaluated for its potential as a predictive biomarker for anti-EGFR mAb therapy in the patients without mutations in *KRAS* with operable colorectal liver metastases (Pugh et al., 2017).

In an interventional clinical trial in 1,808 subjects (ClinicalTrials.gov, Trial Registration ID: NCT03362684), the predictive potential of the miR-31-3p expression level was studied for the prognostic of patient outcomes, as was the predictive value of the benefit of anti-EGFR therapy (cetuximab) in stage III CRC patients (the patients enrolled in the PETACC-8 Study) (Taieb et al., 2014). The results obtained from this clinical trial demonstrated that patients with the RAS/BRAF wild type who showed low miR-31-3p expression when tumors were treated with cetuximab plus FOLFOX-4 presented improved disease-free survival, overall survival, and survival after recurrence compared with the patients treated with FOLFOX-4 alone.

More recently with logistic regression models, including the miR-31-3p expression level adjusted for potential confounding factors, Laurent-Puig et al. (2019) validate the use of miR-31-3p to differentiate RAS wt metastatic CRC patient outcomes from patients treated with anti-EGFR mAb or anti-VEGF mAb therapy. Those patients with low miR-31-3p levels showed better outcomes when treated with cetuximab compared with bevacizumab.

The miRpredX-31-3p kit was developed on the basis of a standardized RT-qPCR assay for miRNA detection. Several extraction kits (miRNeasy FFPE kit (Qiagen), AllPrep DNA/RNA FFPE Kit (Qiagen), QIAsymphony RNA kit (Qiagen), and Maxwell 16 LEV RNA FFPE kit (Promega)) have been tested to evaluate the efficiency of miRNA extraction from five formalin-fixed, paraffin-embedded (FFPE) 5-mm-thick slides. In addition, the analytical sensitivity and specificity, assay robustness, reproducibility, and accuracy of miR-31-3p detection were also demonstrated in different quantitative PCR systems like ABI 7900HT^®^, ABI StepOne+^®^, and ABI QS5^®^ (Applied Biosystems) and LightCycler^®^ 480 (Roche) (Ramon et al., 2018). These results demonstrated the good versatility of the miRpredX-31-3p assay and its feasibility for being easily implemented into clinical diagnostic laboratories. The time to perform the assay was not as long after total RNA was isolated from FFPE tumor samples because the assay is based on a simple RT-qPCR reaction (reverse transcription and subsequent real-time PCR). Hence the miRpredX-31-3p assay can analyze up to 12 samples and provide the results in 1 day (see version 8 of the mirpredx instructions manual).

#### The Nu.Q^™^ Colorectal Cancer Screening Triage Test

NuQ^®^ tests (Volition SA; Namur, Belgium) are intended for diagnosing CRC by analyzing different nucleosome characteristics, including the DNA methylation of DNA bound to nucleosomes, post-translational modifications in histones and histone variants, and the detection of cell-free nucleosomes, although the company is developing a new test based on these biomarkers. NuQ^®^ tests are based on Enzyme-linked immunosorbent assay (ELISA) technology and require only one drop of blood from patients (a 10-μl sample).

The most advanced test is the Nu.Q^™^ Colorectal Cancer Screening Triage Test, which consists in combining different NuQ^®^ previously CE-IVD marked tests. One of them is the NuQ^®^X test, which detects the 5-methylcytosine levels present in DNA bound to cell-free circulating nucleosomes.

In a validation study performed by Holdenrieder et al. (2014), serum samples were used in two independent cohorts of subjects: i) 90 subjects, including CRC patients (n = 24), benign colorectal diseases (BCD) (n = 10), and healthy controls (n = 56); ii) 113 subjects, including CRC patients (n = 49), BCD (n = 26), and healthy controls (n = 38). Holdenrieder et al. (2014) used the Nu.Q^®^X test to evaluate its differential diagnostic performance. Their study showed that the circulating methylated DNA levels significantly lowered in CRC and BCD compared with the healthy controls (p < 0.05), although no difference was found between BCD and CRC. The AUC on the receiver operating characteristic curve was 0.78, and sensitivity was 33% at 95% specificity for CRC and BCD compared to HC, with a sensitivity of 75% at 70% specificity for CRC compared to HC.

To improve both the sensitivity and specificity of the assays, two new tests were designed: the Nu.Q^®^T test and the NuQ^®^V test. Both obtained the CE-IVD mark. The Nu.Q^®^T test was designed for the diagnostic of CRC by detecting total free circulating nucleosomes (cell-free nucleosome). Nu.Q^®^V focused on detecting CRC by analyzing histone variants. Finally, they were included in a NuQ^®^ test based on the same nucleosomics ELISA technology (Holdenrieder et al., 2014).

Rahier et al. (2017) used the Nu.Q^®^ assay to evaluate the levels of 12 epitopes [including nucleosome-associated histone modifications: H4K20me3 (mAb), H4PanAc (mAb), pH2AX (mAb), H3K9Me3 (pAb), H2AK119Ub (mAb), H3K9Ac (mAb), and H3K27Ac (mAb); nucleosome-associated DNA modification: 5mC (mAb); nucleosome containing histone variants: H2AZ (mAb); nucleosome-protein adducts: HMGB1 (mAb) and EZH2 (mAb); and finally a conserved nucleosome epitope as reference of total nucleosome content] in the sera of 58 individuals referred for endoscopic CRC detection [patients with CRC (n = 23), patients with pre-cancerous lesions (polyps) (n = 16), and healthy controls (n = 19)]. The multivariate analysis defined a panel of four age-adjusted cf-nucleosomes that provided an AUC of 0.97 for the CRC discrimination of healthy controls with high sensitivity in initial stages (sensitivity of 75% and 86% and specificity of 90% for stages I and II, respectively). The second combination of four cf-nucleosome biomarkers provided an AUC of 0.72 for the identification of patients with pre-cancerous lesions (polyps) (n = 16) in healthy subjects (Rahier et al., 2017).

The Nu.Q^™^ Colorectal Cancer Screening Triage Test, which is based on the previous described Nu.Q^®^ tests, was evaluated in blinded serum samples from 1,961 FIT-positive individuals. In a set of samples “training set” (n = 1,907), the Nu.Q^™^ Colorectal Cancer Screening Triage test had the potential to identify a subset of 477 subjects in which colonoscopy was applied and could be avoided. Moreover, the test detected 96.6% of CRCs and 88.5% of high-risk adenomas. The results were corroborated in the “validation set” of samples (n = 1,961), which gave a sensitivity of 91.2% for CRC and 83.0% for high-risk adenoma. Ii was noteworthy that the sensitivity for “screen relevant neoplasia” (considering patients with CRC and high-risk adenomas) was about 86% compared with the 80% obtained with positive FIT and a cutoff value of 200 ng/ml. The results of this large cohort evaluation were promising as the Nu.Q^™^ Colorectal Cancer Screening Test can reduce unnecessary colonoscopies by 20%, while maintaining sensitivity for CRC close to 90% (Marielle et al., 2017).

The Volition Company announced that the new Nu.Q^™^ assay would have the potential to detect 81% of CRCs with a specificity of 78% in a cohort of 4,800 CRC patients. Furthermore, the new Nu.Q^™^ assay detected up to 67% of high-risk adenomas with a specificity of 80% in a cohort of 530 symptomatic patients and initial stage I cancers with a sensitivity of 74% and a specificity of 90% in a pilot study of 58 asymptomatic patients. However, we were unable to find any published results or any registered clinical trial results of this study apart from the company’s published interim results.

The Volition Company is developing new-generation Nu.Q assays for other intended uses, such as pancreatic cancer. In fact the Nu.Q assay was also evaluated for the diagnostic of pancreatic cancer. By using a combination of carbohydrate antigen 19-9 (CA 19-9) levels with a panel of four cf-nucleosome markers, Bauden et al. (2015) obtained an AUC of 0.98 with an overall sensitivity of 92% at a 90% specificity to detect pancreatic cancer in serum samples from a cohort of 59 subjects [including patients with resectable pancreatic cancer (n = 25), patients with benign pancreatic disease (n 0), and healthy individuals (n = 24)].

### An Epigenetic-Based IVD Test for Breast Cancer

Breast cancer (MIM 114480) is the most commonly diagnosed cancer in women (Torre et al., 2017) and the leading cause of death from cancer in women worldwide (Torre et al., 2016). It is noteworthy that breast cancer can also affect men and, consequently, around 2,670 new cases of invasive breast cancer are expected to be diagnosed in men in 2019. About 20% of breast cancers worldwide are due to environmental or lifestyle risk factors, such as alcohol abuse, excess body weight and fat, and a sedentary lifestyle (Danaei et al., 2005). In addition, screening with the mammography technique has demonstrated its ability to detect breast cancer in early stages, which reduces the mortality risk and increases treatment success (Lauby-Secretan et al., 2015). As a result, new methods that contribute to early diagnosis, the identification of specific subtypes, and the selection of patients who can benefit from specific therapies will increase patient survival for this cancer.

Breast cancer mortality rates are higher than those for any other cancer and account for 25% of cancer cases and 15% of cancer-related deaths (Ferlay et al., 2015). Breast cancer mortality also depends on the cancer subtype. Breast cancer presents several classifications depending on different aspects. It can be classified according to their histological origin, cell differentiation degree, stage, the presence or absence of certain hormone receptors [i.e., hormonal receptors, like estrogen receptor (ER), and progesterone receptor (PR); and the ERBB2 receptor], and molecular subtype (i.e., luminal A, luminal B, HER2, basal-like subtype, normal-like subtype, and Claudin-low subtype).

Tumors classified as triple-negative breast cancer (TNBC) and HER2-positive breast cancer are classified as high-risk cancer with a poor prognosis (Harbeck and Gnant, 2017). Enhancing breast cancer survival and outcome by early detection remains one of the main breast cancer priorities according to the World Health Organization (WHO). Therefore, several efforts are being made by the research community to provide not only new drugs and therapies to treat breast cancer, but to also identify new biomarkers to help implement precision medicine into the clinical management of breast cancer patients (Low et al., 2018). Breast cancer treatment depends partially on the disease state and the breast cancer subtype. Generally speaking, the commonest treatments are targeted therapy, hormonal therapy, radiation therapy, surgery, and chemotherapy, although immunotherapy is being increasingly utilized. Fortunately, the therapeutic options for breast cancer patients are further improved thanks to the use of biomarkers and the implementation of precision medicine (Meisel et al., 2018), in which epigenetic biomarkers can further improve the battery of *in vitro* assays to manage breast cancer.

#### The Therascreen PITX2 RGQ PCR Kit

The *Therascreen PITX2 RGQ PCR kit* (Qiagen, Germany) is a methylation-based CE-IVD marked assay that predicts the response of lymph node-positive, ER-positive, and HER2-negative high-risk breast cancer patients. The test differentiates between the patients more likely to respond to anthracyclines chemotherapy (Aubele et al., 2017), and it obtained the CE-IVD mark in 2018.

The methylation analysis of *PITX2* (a promoter of transcription factor 2 of the pituitary homeobox) demonstrates a high correlation with other diagnostic techniques, has the predictive and prognostic capability for patient identification, and supports clinicians by being the most effective therapy option. *PITX2* methylation has attracted the attention of clinicians for not only breast cancer (Widschwendter et al., 2004; Aubele et al., 2017), but also for other tumor types. Continuous scientific evidence indicates the potential of the *PITX2* methylation analysis to predict breast cancer outcomes in lymph node-positive, ER-positive, and HER2-negative breast cancer patients to adjuvant anthracycline-based chemotherapy. Therefore, these clinical observations reinforce the idea of using *PITX2* methylation status to support clinicians as the most effective therapy option (Hartmann et al., 2009; Absmaier et al., 2018).

Hartmann et al. (2009) showed that the *PITX2* DNA methylation improved the prediction by using only clinical factors like tumor stage, grade, or age in a cohort of >200 patients. *PITX2* plays an essential role in the disease pathogenesis. In fact, tumors with a hypermethylated *PITX2* status correlate with poorer survival (overall survival and reduced metastasis-free survival), and also with resistance to treatment. In addition, *PITX2* methylation has been associated with the response to adjuvant chemotherapy (Absmaier et al., 2018; Sheng et al., 2017).

Absmaier et al. (2018) explored the validity of this new predictive candidate biomarker in a retrospective exploratory study. To do so, these authors determined the *PITX2* DNA methylation status in non-metastatic TNBC patients treated with adjuvant chemotherapy with anthracycline by a molecular analysis of breast cancer tissues. Univariate and multivariate analyses demonstrated the statistically independent predictive value of *PITX2* DNA methylation. The authors concluded that for those patients with non-metastatic TNBC, the selective determination of the *PITX2* DNA methylation status can serve as a cancer biomarker to predict responses to anthracycline-based adjuvant chemotherapy (Absmaier et al., 2018).

Schriker et al. (2018) performed a clinical study to analyze the performance of the *PITX2* DNA methylation assay compared to microarray technology. These authors concluded that the performance of the *Therascreen PITX2 RGQ PCR* assay showed high reliability and robustness to predict the outcome of patients with high-risk breast cancer to anthracycline-based chemotherapy. In this study, three CpGs from the *PITX2* promoter 2 gene (*PITX2P2*; 4q25) contained in the methylation array (Maier et al., 2007) were selected, and the appropriate Taqman probes were designed to cover these three CpGs in the Therascreen PITX2 RGQ assay (Schricker et al., 2018).

The *Therascreen PITX2 RGQ PCR assay*, developed by Perkins et al. (2018) in conjunction with the Therawis Diagnostics Company, analyzes the methylation status of the *PITX2* gene obtained from the DNA of FFPE biospecimens. *PITX2* methylation is assessed by methylation-specific real-time PCR and exploits the quantitative PCR (qPCR) oligonucleotide hydrolysis principle of two TaqMan probes labeled with different fluorescent dies (FAM^™^ for fully methylated and HEX^™^ for fully unmethylated DNA) in combination with methylation nonspecific primers to measure the methylation status of the target sequences of PITX2 gene promoter 2 in bisulfite-treated DNA. The *Therascreen PITX2 RGQ PCR kit* (Qiagen, Catalog no. 873211) has been currently CE-IVD marked and is commercially available (Aubele et al., 2017; Schricker et al., 2018). It runs in the real-time Rotor-Gene Q MDx thermal cycler (Qiagen) or a Rotor-Gene Q MDx 5plex HRM instrument (Qiagen). The percentage of the methylation ratio (PMR  =  100/(1 + 2exp(CtFAM(methylated) − CtHEX(unmethylated))]) is calculated by the Rotor-Gene AssayManager^®^ software with a Gamma Plug-in plus a kit-specific *PITX2* Assay Profile for automated analyses and quality control, including all the validity criteria. Detailed information about the method is described by Schricker et al. (2018) and Maier et al. (2007). The *Therascreeen PITX2 RGQ PCR assay* can be easily adopted in clinical laboratories that already run other Therascreen assays commercialized by Qiagen. The complete workflow is streamlined throughput for a medium sample with highly reliable and robust readouts and can be performed in a time of 2 working days (Perkins et al., 2018).

### The Epigenetic-Based IVD Test for Cervical Cancer

Cervical cancer (MIM 603956) is the fourth most frequent cancer in women, with an estimation of 570,000 new cases in 2018, which represents 6.6% of all female cancers. Cervical cancer is the fourth commonest cause of death from cancer in women (Vu et al., 2018), which is approximately 8% of the total deaths from cancer. Furthermore, as cervical cancer has no shown symptoms in its early stages, early identification of cervical precancerous lesions is of critical importance (Gradíssimo and Burk, 2017).

More than 90% of cases are due to infection with human papillomavirus (HPV) (Kumar et al., 2007; Crosbie et al., 2013), and despite people having had HPV infections and them not developing cervical cancer (Dunne and Park, 2013), organized vaccination and screening programs are essential to lower the cervical cancer incidence (Vu et al., 2018).

Thus, cytology-based screening is widespread and has proven to effectively lower cervical cancer incidence rates in many countries (Anttila and Nieminen, 2000). However, the relatively low sensitivity of a single Pap smear and the higher false-negative results, and sometimes the requirement of multiple Pap tests, make cytology-based screening costs prohibitive for the early identification of precancerous lesions. Therefore, preventive programs focus on HPV testing as a primary screening tool for the early detection of the causative agent of cervical cancer (Dillner et al., 2008). In fact, primary high-risk HPV (hrHPV) screening has recently become an accepted stand-alone or co-test with conventional cytology in preventive cervical cancer programs.

Chang et al. (2015) found that several genes, such as *PAX1*, *ZNF582*, and *SOX1*, were hypermethylated in cervical cancer compared to normal cervical tissue. Shen-Gunter et al. (2016) evaluated the performance of analyzing the HPV genotype and measuring DNA methylation at promoters *ADCY8*, *CDH8*, and *ZNF582* correlated with the cytological grade, therefore demonstrating their potential to be useful biomarkers for the molecular classification of Pap smears. With their systematic literature review, Wentzensen et al. (2009) attempted to identify promising methylation-based biomarkers for the early detection of cervical cancer. These authors found that the elevated methylation of *DAPK1*, *CADM1*, and *RARB* in cervical cancer was consistently observed in several studies and thus became interesting candidates to be validated in large cohorts during standardized clinical trials (Wentzensen et al., 2009). However, no consensus has been reached about which promoter or gene methylation should be analyzed, and whether these will develop into molecule tests with sufficient predictive values or be useful for the early detection of precancerous lesions. One epigenetic test, based on the analysis of the methylation of genes *ZNF582* and *PAX1*, is being commercialized.

#### The Cervi-M^®^ and Oral-M^®^ DNA assays

The Cervi-M^®^ and Oral-M^®^ DNA assays (by Epigene, iStat Biomedical Co.; Taiwan) obtained CE-IVD approval for the diagnostic of cervical and oral cancers. iStat Biomedical Co. commercializes the Cervi-M^®^, *ZNF582* DNA, and the Oral-M^®^ assay, which are based on the methylation analysis of *genes ZNF582* and *PAX1*. These genes are highly methylated in cervical and oral cancers, as described by Lin et al. (2014) and Chang et al. (2015). Gene *ZNF582* codifies for zinc finger protein 582, which plays a key role in transcriptional regulation. *ZNF582* methylation status has been demonstrated as a good biomarker for cervical cancer induced by HPV, with a sensitivity of 73% and a specificity of 80% (Lin et al., 2014). Furthermore, *ZNF582* methylation status shows high sensitivity for the detection of grade-3 cervical intraepithelial neoplasia or in a higher stage (CIN3+) (Liou et al., 2016), and demonstrates its utility to improve diagnostic accuracy more than single HPV DNA testing (Li et al., 2019). In addition, the *PAX1* DNA methylation assay allows the detection of cervical cancers graded as CIN3+, as described by Lai et al. (2008) and Lai et al. (2010). This assay generates clinical sensitivity and specificity above 80% when used with the DNA purified from Pap smears (data provided by the company).

The ZNF582/PAX1 assay consists of the bisulfite treatment of DNA obtained from human epithelial cells collected by cervical brush. Then 20 to 80 ng of bisulfite-converted DNA is analyzed by methyl-specific quantitative PCR in a LightCycler^®^ 480 Instrument (Roche) or an Applied Biosystems^®^ 7500 fast system following the protocol described by the manufacturer (see the instructions in the manual). As the analysis depends on the kit used for the bisulfite treatment of DNA, which can last up to 1 day, the complete workflow to perform the Cervi-M^®^ and Oral-M^®^ DNA assays takes about 2 working days.

It is interesting to note that although the Cervi-M^®^ assay has been tested only in the DNA obtained from epithelial cells collected by cervical brush, as the female reproductive tract and regular uterine endometria shedding into the vagina may exfoliate cells, Bakkum-Gamez et al. (2015) proposed using vaginal tampons as a source of DNA to detect endometrial cancer by an assay of methylated DNA markers.

### The Epigenetic-Based IVD Test for Glioblastoma

Glioblastoma (GBM, MIM 137800) is the most common primary malignant brain tumor in adults with an unfavorable prognosis and limited treatment options despite innovative diagnostic strategies and new therapies having been developed (Lombardi and Assem, 2017). GBM constitutes approximately 45% to 50% of all primary malignant brain tumors and is diagnosed more frequently in patients aged between 55 and 85 years, with a mean age of 64 years in the United States (Louis et al., 2016). Evidence in recent years has demonstrated that tumors are made of multiple populations of cancerous cells by harboring specific genetic alterations in addition to the classic founder genetic abnormalities and epigenetic alterations that drive intratumor heterogeneity with multiple different cell subpopulations (Gerlinger and Swanton, 2010; Lombardi and Assem, 2017).

*EGFR* amplification, *IDH1/2* mutations, and *MGMT* promoter methylation have been proposed as prognostic biomarkers for their molecular and clinical significance. *MGMT* promoter methylation is one of the most relevant prognostic markers and can be used to also predict the therapeutic response to one of the therapeutic strategies for GBM based on the use of alkylating agents like carmustine (BCNU, Gliadel^®^) and temozolomide (Temodar^®^). This is because *MGMT*, an O6-methylguanine-DNA-methyltransferase, is a DNA-repairing gene whose silencing may increase the susceptibility of cells to temozolomide concurrently with radiation therapy (Zawlik et al., 2009). Furthermore, increased methylation of the *MGMT* promoter measured by pyrosequencing has been related to increased GBM patient survival (Zhao et al., 2016).

#### The PyroMark Therascreen MGMT Kit and the PyroMark Q96 CpG MGMT Kit

The *MGMT* methylated status is a strong predictor of the response to temozolomide in patients with GBM during therapy with alkylating agents. Therefore, the DNA methylation of this gene has been postulated as a biomarker to classify gliomas and to guide treatment decision-making (Gusyatiner and Hegi, 2018).

Quillien et al. (2012) found that pyrosequencing led to the highest reproducibility and sensitivity in *MGMT* methylation status analyses, as was also confirmed by Hsu et al. (2017) after testing four different techniques (e.g., immunohistochemistry, MSP, qMSP, and pyrosequencing) to analyze the *MGMT* methylation status. Different commercialized kits are available for the pyrosequencing methodology, such as the PyroMark Q96 CpG MGMT kit (cat. number 972032; Qiagen), which uses the PyroMark Q96 MD system (Qiagen), and the test Therascreen MGMT PyroKit (cat. number 972032; Qiagen), which uses the pyrosequencing PyroMark Q24 system (Qiagen) with specific sequencing primers. The PyroMark Q96 CpG MGMT kit detects five CpG sites located in exon 1 (CpG 74–78), whereas the CE-IVD commercialized kit, the PyroMark Therascreen MGMT kit, detects four CpG sites in the same location (CpG 76–79) of the human *MGMT* gene in DNA samples obtained from blood or FFPE biospecimens. Briefly, the assay consists of using bisulfite converted genomic DNA (with the EpiTect Bisulfite kit, cat. number 59104; Qiagen) for subsequent PCR amplification to sequencing it by pyrosequencing using the kits and systems described above to analyze the methylation status of exon 1 of the *MGMT* gene. The sequences surrounding the defined positions serve as normalization and reference peaks for the quantification and quality assessment of the analysis (see the manufacturer’s instructions). The time it takes to obtain results relies on the bisulfite treatment of DNA, which needs 6 to 8 h to complete the workflow of the *MGMT *methylation status analysis and lasts about 2 working days.

After performing the PCR using primers by targeting the defined region of exon 1, amplicons are immobilized on Streptavidin Sepharose High Performance beads. Then single-stranded DNA is prepared, and the sequencing primers are annealed to DNA. Samples are then analyzed in the PyroMark Q24 system.

Both kits (PyroMark Q24 CpG MGMT and Therascreen MGMT PyroKit) have demonstrated their capability to stratify patients with GBM according to its prognostic after measuring *MGMT* promoter methylation (Johannessen et al., 2018). Quillien et al. (2017) evaluated the ability of the Therascreen MGMT kit in 102 glioblastoma patients and found that using a binary classification of methylated/unmethylated *MGMT* gene with cutoffs of 8% and 12%, 95% and 97% of GBM patients were well classified. Quillien et al. (2017) also found an excellent prognostic capability of the assay and indicated median overall survival of 15.9 and 34.9 months, respectively, for unmethylated and methylated patients. Moreover, the use of the *MGMT* methylated status as a predictor of meningioma has been recently tested by Panagopoulos et al., but these authors concluded that the methylation frequency of the *MGMT* promoter in meningioma is very low (6%) and, therefore, suggested that *Therascreen MGMT PyroKit* is not suitable for meningiomas.

As *MGMT* is methylated to 25% to 50% in numerous cancers, including brain, colon, lung, breast, gastric, and ovarian cancer (Gerson, 2004), it involves the risk of offering positive results for cancer patients who were found negative for GBM.

### The Epigenetic-Based IVD Test for Lung Cancer

Lung cancer (MIM 211980) is the leading cause of death from cancer worldwide (Siegel et al., 2017), and 8 or 9 of 10 lung cancer cases occur in smokers. Thus, smoking is the biggest risk factor of this disease. The 5-year survival rate after diagnosis is 15.6%, which is lower than the survival rates for breast, colon, and prostate cancers. The WHO classifies lung cancer into two broad histological subtypes. The first one is non–small-cell lung cancer (NSCLC), which causes about 85% of cases, including lung squamous carcinoma (LUSC), lung adenocarcinoma (LUAD), and large cell carcinoma subtypes. The second subtype is small-cell lung cancer (SCLC), which accounts for the remaining 15% (Couraud et al., 2012).

The treatment that includes surgical, medical, and radiotherapeutic interventions did not much improve the long-term survival rate of those patients diagnosed with primary lung neoplasms. Moreover, classic cisplatin-based chemotherapy for NSCLC, which can be combined with anti-angiogenic bevacizumab, gives low to moderate satisfactory results. The use of specific tyrosine kinase inhibitors (TKi) in *EGFR*-mutated, ALK/ROS1-rearranged NLSC, and the development of new immunotherapy strategies based on anti-PD1/PD-L1 mAb are currently improving the clinical outcomes of lung cancer patients (Duruisseaux and Esteller, 2018). Yet despite new therapies having been designed and applied, tumor resistance to treatments mean that about 154,050 people died from lung cancer in 2018 only in the United States (https://www.cancer.org/). To increase survival rates in lung cancer, early diagnosis is a priority. However, one of the most widely used techniques is the computed tomography (CT) of the thorax and bronchoscopy. CT gives rise to false positives in lung-cancer free patients, delays lung cancer diagnosis, and also exposes these subjects unnecessarily to radiation. Bronchoscopy fails in about half those diagnosed with lung cancer. Therefore, a diagnostic test based on the biological material obtained from non-invasive or minimally invasive samples with high specificity may cut the need for more costly invasive diagnostic procedures.

The current hypothesis to explain lung carcinogenesis considers that tumor development occurs in a multistage stepwise manner that contributes to the accumulation of genetic and epigenetic alterations (Lantuéjoul et al., 2009). Therefore, epigenetic signatures based on dysregulated DNA methylation differentially express miRNA, and altered post-translational modified histones can reflect the driving force of lung carcinogenesis. Accordingly, given the pivotal role of epigenetic disruption during this process, the epigenomic marks detected in tissue or body fluids represent a feasible biomarker to identify disease in its early stages, establish a prognostic, and monitor treatment response (Bhargava et al., 2018). In a recent relevant work, Duruisseaux and Esteller (2018) describe several epigenetic mechanisms that underlie the acquisition of the cancerous phenotype and the aggressive behavior of lung cancer. They also propose circulating epigenetic biomarkers and the therapeutic potential of epigenetic drugs to implement precision medicine in lung cancer.

#### The Epi proLung BL Reflex Assay®

*SHOX2*, or short stature homeobox gene two, methylation has been identified as a biomarker capable of reliably differentiating between lung tumor tissue and normal tissues (Lewin et al., 2007; Schmidt et al., 2010).

*SHOX2* methylation, as determined from bronchial aspirates, has demonstrated good sensitivity and a high specificity as a biomarker for lung cancer (Dietrich et al., 2012b). *Epigenomics AG* commercializes the Epi proLung BL Reflex Assay^®^ (Epigenomics AG, Berlin, Germany), a CE-IVD test for quantifying *SHOX2* methylation using methyl-specific PCR with AUC [95% confidence intervals] = 0.94 [0.91–0.98], sensitivity 78% [69–86%], and specificity 96% [90–99%] in bronchial lavage specimens (Dietrich et al., 2012b), albeit with lower sensitivity (about 40%) in malignant pleural effusions (Ilse et al).

The Epi proLung BL Reflex Assay^®^ is composed of three individual kits: The Epi proLung BL DNA Preparation Kit to prepare bisulfite converted DNA by ammonium bisulfite chemistry, the Epi proLung BL real-time PCR Kit for the quantitative and sensitive analyses of the relative amount of methylated *SHOX2* gene, and the Epi proLung BL Work Flow Control Kit for monitoring and controlling the whole workflow. A detailed explanation of the different steps performed in the *SHOX2* gene methylation analysis using the Epi proLung BL Reflex assay^®^ is described by Dietrich et al. (2012a). Like other methylation-based assays, the time to obtain the results relies on the bisulfite treatment of DNA, which requires about 8 h. Therefore, 2 working days are needed to complete the workflow of the Epi proLung BL Reflex Assay^®^.

In 2011, *SHOX2* methylation was assessed in circulating cell-free DNA obtained from blood plasma and showed a sensitivity of 60% and a specificity of 90% for lung cancer diagnosis in a case-control study with 343 subjects (Kneip et al., 2011). Since then, Epigenomics AG has been working on demonstrating the test’s utility. In 2017, the Epi proLung^®^ blood-based version for the lung cancer test received the CE-IVD mark, which is based on a combination of the methylation analyses of *SHOX2* and *PTGER4* (the prostaglandin E receptor 4 gene). In fact, Weiss et al. (2017) demonstrated significant discriminatory performance for distinguishing patients with lung cancer from subjects with no malignancy (AUC [95% confidence intervals] = 0.88, sensitivity 90%, and specificity 73%) in circulating DNA from plasma samples by the methylation analysis of genes *SHOX2* and *PTGER4*.

The current commercial Epi proLung^®^ assay consists of the Epi proLung PCR Kit (M6-02-002) and the Epi proLung Control Kit (M6-02-003), and has been validated with bisulfited-treated DNA prepared with the Epigenomics Epi BiSKit (M7-01-001) from cell free-circulating DNA present and isolated from 3.5 ml of plasma. The methylation of the *ACTB* gene (ß-actin) is measured as an internal control to assess input adequacy. It also provides positive and negative controls for each run by starting with DNA extraction from plasma. Two methylated *SHOX2*- and *PTGER4*-specific fluorescent detection probes are used in this MethyLight-based assay to exclusively identify the methylated target sequences amplified during the PCR reaction. The assay, with an area under the ROC curve (AUC = 0.82), displays the observed likelihood of being diagnosed with lung cancer according to the EPLT score (ranging from threshold −0.43 to −1.85), together with the corresponding sensitivity (59% to 85%, respectively) and specificity (95% to 50%, respectively), which depends on a given specific threshold (see the Epi proLung^®^ instruction manual for more details)

Epigenomics has performed experiments to evaluate the cross-reactivity of the Epi proLung^®^ assay. Both BLAST alignment searches and PCR analyses against the human genome with the Epi proLung PCR assay (blockers, primers, and probes) have been performed. This analysis showed that the test is specific and only gives the amplification of the bisulfite-treated DNA sequence of methylated *SHOX2* and *PTGER4*, respectively, and not the other regions in the human genome. Epi proLung^®^ was also checked to evaluate the methylated status of *SHOX2* and *PTGER4* in the patients affected by other lung-associated diseases. Fifty-seven (57) samples from patients with non-malignant lung diseases [Chronic obstructive pulmonary disease (COPD), pneumonia, lung emphysema, interstitial lung disease] were evaluated to determine cross-reactivity. The Epi proLung^®^ test discriminated malignant disease from non-malignant disease with an AUC of 0.73.

### The Epigenetic-Based IVD Test for Cancers of Unknown Origin

Cancer of an unknown primary site (CUP) is a heterogeneous group of cancers for which the anatomical site of origin remains hidden after detailed clinical and histological investigations (Briasoulis et al., 2005; Varadhachary and Raber, 2014). CUP is clinically characterized as an aggressive disease with early dissemination (Pentheroudakis et al., 2013) that contributes to their presentation (Varadhachary and Raber, 2014). CUP accounts for 3% to 5% of all cancer diagnoses and is the third commonest cause of death from cancer because, unfortunately, most patients (80–85%) do not respond appropriately to treatment (Pavlidis and Fizazi, 2009; Pavlidis and Pentheroudakis, 2012). Therefore, patient survival is very limited.

Tumors in CUP share biologic and molecular properties, but tumors in CUP are currently indicated to maintain the signature of the putative primary origin. The general characteristics of CUP are: 1) short natural history with symptoms and signs associated with metastatic sites; 2) early rapid dissemination in the absence of a primary tumor (three organs or more are involved upon diagnosis in one third of patients); 3) aggressive clinical progression; and 4) sometimes an unpredictable metastatic pattern that differs from those of known primary tumors (Pavlidis and Fizazi, 2009; Pavlidis and Pentheroudakis, 2012).

The heterogeneous CUP presentations mean that immunohistochemical testing, the characterization of tissue-of-origin molecular profiling, and the assignation of appropriate therapies present a challenge (Varadhachary and Raber, 2014). Classifying CUP patients into several clinicopathological subsets is necessary for oncologists to manage these patients and to decide about appropriate therapies. This classification is done according to socio-demographic criteria, such as age and gender, histopathology patterns, clinico-pathological data, laboratory tests, and image data (MRI, PET, CT scanning, mammography, etc.), and also to the affected organ or site. Despite several immunohistochemistry panels having been developed to diagnose CUP, the primary cancer site remains unknown in about 75% of patients (Varadhachary and Raber, 2014). Therefore, the need to find new diagnostic tools to discover the tissue of origin is substantial.

#### EPICUP^™^

EPICUP^™^ (Ferrer, Spain) is a CE-IVD test used to biologically define the tissue of origin in CUP. EPICUP^™^ was the first epigenetic test designed to identify tumors of unknown primary and claims that it can identify up to 87% of cases of cancer of unknown origin (Moran et al., 2016). The EPICUP^™^ test is based on the analysis of 485,577 CpG sites measured by the human methylation matrix Infinium HumanMethylation450 Beadchip microarray (Illumina), and the test was designed to look for similarities in the methylation patterns of cancers of unknown primary and known primary tumors. Based on the results, the EPICUP^™^ test is able to perform an epigenetic identification and subsequent categorization of the primary site in CUP cancers from FFPE or frozen tissue samples (Moran et al., 2016). This is not a suitable assay for all clinical laboratories because the EPICUP^™^ test is based on Illumina methylation BeadChip. Therefore, the mean time to provide results takes about 2 weeks if it is to consider DNA purification from tissue, the bisulfite treatment of purified DNA, array hybridization, and, finally, bioinformatic data analyses and their interpretations.

EPICUP^™^ classifies the tumor type based on the study of DNA methylation profiles using the Infinium HumanMethylation450 Beadchip microarray DNA methylation signature. It offers a specificity of 99.6%, a sensitivity of 97.7%, a positive predictive value of 88.6%, and a negative predictive value of 99.9% in a validation set of 7,691 tumors. Thus, with the samples of 216 CUP patients (FFPE tissue), the DNA methylation profile was able to predict a cancer of primary origin in 188 patients (87%) (Moran et al., 2016).

EPICUP^™^ demonstrates its ability to provide the correct treatment to CUP patients. In fact, the patients who received tumor-specific therapy diagnosed with EPICUP showed better overall survival than those who received empirical therapy [hazard ratio (HR) 3.24, p = 0.0051 (95% CI, 1.42–7.38); log-rank p = 0.0029] (Moran et al., 2016). Likewise, EPICUP in a study of DNA methylation profiles was proven a cost-effective test in breast, pancreas, colon, lung (NSCLC), and prostate cancers and increased the overall survival adjusted for quality (Gracia et al., 2015).

## Conclusions

Modern medicine moves toward more personalized practice and theragnosis, and epigenetic biomarkers can further contribute to all of this. This review describes the most advanced and commercially available tests based on epigenetic biomarkers that help to improve precision medicine. In some cancers, such as CRC, several options are available, based on stool DNA (i.e., Cologuard^®^ and EarlyTect^®^), liquid biopsy (Epi ProColon^®^, EarlyTect^®^ and NuQ^™^), and FFPE (miRPredX-31-3p). Other tests, such as the Therascreen MGMT Pyro kit for glioblastoma, can be used in the DNA obtained from blood and FFPE tissues. Obviously, for clinical settings and to avoid invasive procedures, tests based on a liquid biopsy are preferable.

Methodologically speaking, to implement these new epigenetic tests into clinical routine, most of these tests have adopted easy-to-use inexpensive analytical methods, like those based on RT-qPCR and microarrays for both DNA methylation and miRNA analyses. There is still a long way ahead before these epigenetic tests can be completely implemented into clinical routine. The companies developing epigenetic tests should focus their efforts on simplifying the technology used to analyze epigenetic biomarkers in a clinical laboratory environment by, for example, using qPCR-based technology, which is easy to use and cost-effective. Moreover, companies have to make efforts to identify biomarkers in non-invasive biospecimens, which will contribute to anticipate cancer diagnosis and to also increase patient compliance with screening campaigns.

We are witnessing a revolution by adapting machine learning procedures to epigenetic biomarkers analyses that will contribute to definitely implement new epigenetic biomarkers into clinical routine. In fact, advanced computational techniques have taken us closer to realize the application of epigenetics to personalized medicine (Holder et al., 2017). One important scenario is that the cost of specific treatments and the appropriate use of targeted therapies guided by epigenetic biomarkers are expected to streamline the immense cost required to receive personalized therapies.

## Author Contributions

JB-G, RO-V, SM-M, and JLG-G contributed to bibliography compilation and analysis. JB-G, RO-V, SM-M, and JLG-G contributed to manuscript drafting. JB-G, RO-V, SM-M, and JLG-G reviewed the manuscript content. All authors approved the final version of the manuscript.

## Funding

This work was supported by a 2017 VLC-Bioclinics grant and the Generalitat Valenciana (GV/2014/132), AES2016 (ISCIII) with grant number PI16/01036 and Proyectos de Desarrollo Tecnológico en Salud with grant number DTS17/132 AES2017, co-financed by the European Regional Development Fund (ERDF), Instituto de Salud Carlos III through CIBERer (Biomedical Network Research Center for Rare Diseases and INGENIO2010). JB-G is supported by a grant Contratos i-PFIS (IFI18/00015) and co-financed by the European Social Fund. RO-V is supported by the grant APOTIP/2019/A/015 “Subvenció per a la Contractació de Personal de Suport vinculat a un projecte d’investigació o de Transferència Tecnològica” Conselleria de Educación, Investigación, Cultura y Deporte de la Generalitat Valenciana.

## Conflict of Interest Statement

JLG-G is the Chief Executive Officer and SM-M is the Chief Scientific Officer of EpiDisease S.L. Both own shares in EpiDisease SL., an epigenetics company that focuses on developing epigenetic biomarkers. The remaining authors declare that the research was conducted in the absence of any commercial or financial relationships that could be construed as a potential conflict of interest.

## References

[B1] Absmaier M. Napieralski R. Schuster T. Aubele M. Walch A. Magdolen V. (2018). PITX2 DNA-methylation predicts response to anthracycline-based adjuvant chemotherapy in triple-negative breast cancer patients. Int. J. Oncol. 52 (3), 755–767. 10.3892/ijo.2018.4241 29328369 PMC5807037

[B2] Ahlquist D. A. (2018). Universal cancer screening: revolutionary, rational, and realizable. npj Precis. Oncol. 2, 23. 10.1038/s41698-018-0066-x 30393772 PMC6206005

[B3] Anttila A. Nieminen P. (2000). Cervical cancer screening programme in Finland. Eur. J. Cancer 36, 2209–2214. 10.1016/S0959-8049(00)00311-7 11072206

[B4] Aubele M. Schmitt M. Napieralski R. Paepke S. Ettl J. Absmaier M. (2017). The predictive value of PITX2 DNA methylation for high-risk breast cancer therapy: current guidelines, medical needs, and challenges. Dis. Markers 2017, 1–14. 10.1155/2017/4934608 PMC5613359 29138528

[B5] Bakkum-Gamez J. N. Wentzensen N. Maurer M. J. Hawthorne K. M. Voss J. S. Kroneman T. N. (2015). Detection of endometrial cancer via molecular analysis of DNA collected with vaginal tampons. Gynecol. Oncol. 137, 14–22. 10.1016/j.ygyno.2015.01.552 25677060 PMC4380654

[B6] Bauden M. Pamart D. Ansari D. Herzog M. Eccleston M. Micallef J. (2015). Circulating nucleosomes as epigenetic biomarkers in pancreatic cancer. Clin. Epigenetics. 7 (1), 106. 10.1186/s13148-015-0139-4 26451166 PMC4597435

[B7] Bhargava A. Bunkar N. Aglawe A. Pandey K. C. Tiwari R. Chaudhury K. (2018). Epigenetic biomarkers for risk assessment of particulate matter associated lung cancer. Curr. Drug Targets 19, 1127–1147. 10.2174/1389450118666170911114342 28891455

[B8] Boleij A. Tack V. Taylor A. Kafatos G. Jenkins-Anderson S. Tembuyser L. (2016). RAS testing practices and RAS mutation prevalence among patients with metastatic colorectal cancer: results from a Europe-wide survey of pathology centres. BMC Cancer 16, 825. 10.1186/s12885-016-2810-3 27784278 PMC5080758

[B9] Briasoulis E. Tolis C. Bergh J. Pavlidis N. ESMO Guidelines Task Force (2005). ESMO Minimum Clinical Recommendations for diagnosis, treatment and follow-up of cancers of unknown primary site (CUP). Ann. Oncol. Off. J. Eur. Soc. Med. Oncol. 16 (Suppl 1), i75–76. 10.1093/annonc/mdi804 15888766

[B10] Chang C.-C. Huang R.-L. Liao Y.-P. Su P.-H. Hsu Y.-W. Wang H.-C. (2015). Concordance analysis of methylation biomarkers detection in self-collected and physician-collected samples in cervical neoplasm. BMC Cancer 15, 418. 10.1186/s12885-015-1411-x 25985991 PMC4448302

[B11] Church T. R. Wandell M. Lofton-Day C. Mongin S. J. Burger M. Payne S. R. (2014). Prospective evaluation of methylated SEPT9 in plasma for detection of asymptomatic colorectal cancer. Gut 63, 317–325. 10.1136/gutjnl-2012-304149 23408352 PMC3913123

[B12] Couraud S. Zalcman G. Milleron B. Morin F. Souquet P.-J. (2012). Lung cancer in never smokers – A review. Eur. J. Cancer 48, 1299–1311. 10.1016/j.ejca.2012.03.007 22464348

[B13] Crosbie E. J. Einstein M. H. Franceschi S. Kitchener H. C. (2013). Human papillomavirus and cervical cancer. Lancet (London, England) 382, 889–899. 10.1016/S0140-6736(13)60022-7 23618600

[B14] Dae Han Y. Jeong Oh T. Chung T.-H. Won Jang H. Nam Kim Y. An S. (2019). Early detection of colorectal cancer based on presence of methylated syndecan-2 (SDC2) in stool DNA. Clin. Epigenetics. 11, 51. 10.1186/s13148-019-0642-0 30876480 PMC6419806

[B15] Danaei G. Vander Hoorn S. Lopez A. D. Murray C. J. L. Ezzati M. Comparative risk assessment collaborating group (Cancers) (2005). Causes of cancer in the world: comparative risk assessment of nine behavioural and environmental risk factors. Lancet (London, England) 366, 1784–1793. 10.1016/S0140-6736(05)67725-2 16298215

[B16] DeVos T. Tetzner R. Model F. Weiss G. Schuster M. Distler J. (2009). Circulating methylated SEPT9 DNA in plasma is a biomarker for colorectal cancer. Clin. Chem. 55, 1337–1346. 10.1373/clinchem.2008.115808 19406918

[B17] Diamandis E. P. (2012). The failure of protein cancer biomarkers to reach the clinic: why, and what can be done to address the problem? BMC Med. 10, 87. 10.1186/1741-7015-10-87 22876833 PMC3425158

[B18] Dietrich D. Kneip C. Raji O. Liloglou T. Seegebarth A. Schlegel T. (2012a). Performance evaluation of the DNA methylation biomarker SHOX2 for the aid in diagnosis of lung cancer based on the analysis of bronchial aspirates. Int. J. Oncol. 40, 825–832. 10.3892/ijo.2011.1264 22108652

[B19] Dietrich D. Liebenberg V. Schmidt B. Fleischhacker M. Kneip C. Kristiansen G. (2012b). Development and performance evaluation of a CE-IVD for measuring SHOX2 DNA methylation in bronchial aspirates for the diagnosis of lung cancer. Lung Cancer 77, S22. 10.1016/j.lungcan.2012.05.036

[B20] Dillner J. Rebolj M. Birembaut P. Petry K.-U. Szarewski A. Munk C. (2008). Long term predictive values of cytology and human papillomavirus testing in cervical cancer screening: joint European cohort study. BMJ 337, a1754. 10.1136/bmj.a1754 18852164 PMC2658827

[B21] Dunne E. F. Park I. U. (2013). HPV and HPV-associated diseases. Infect. Dis. Clin. North Am. 27, 765–778. 10.1016/j.idc.2013.09.001 24275269

[B22] Duruisseaux M. Esteller M. (2018). Lung cancer epigenetics: from knowledge to applications. Semin. Cancer Biol. 51, 116–128. 10.1016/j.semcancer.2017.09.005 28919484

[B23] Faruq O. Vecchione A. (2015). microRNA: diagnostic perspective. Front. Med. 2, 51. 10.3389/fmed.2015.00051 PMC4523054 26284247

[B24] Ferlay J. Soerjomataram I. Dikshit R. Eser S. Mathers C. Rebelo M. (2015). Cancer incidence and mortality worldwide: Sources, methods and major patterns in GLOBOCAN 2012. Int. J. Cancer 136, E359–E386. 10.1002/ijc.29210 25220842

[B25] Food and Drug Administration (2019a). Nucleic Acid Based Test. https://www.fda.gov/medical-devices/vitro-diagnostics/nucleic-acid-based-tests

[B26] Food and Drug Administration (2019b). List of cleared or approved companion diagnostic devices (in vitro and imaging tools). https://www.fda.gov/medical-devices/vitro-diagnostics/list-cleared-or-approved-companion-diagnostic-devices-vitro-and-imaging-tools

[B27] García-Giménez J. L. Mena-Mollá S. Beltrán-García J. Sanchis-Gomar F. (2017a). Challenges in the analysis of epigenetic biomarkers in clinical samples. Clin. Chem. Lab. Med., 55 (10), 1–4. 10.1515/cclm-2016-1162 28301317

[B28] García-Giménez J. L. Seco-Cervera M. Tollefsbol T. O. Romá-Mateo C. Peiró-Chova L. Lapunzina P. (2017b). Epigenetic biomarkers: current strategies and future challenges for their use in the clinical laboratory. Crit. Rev. Clin. Lab. Sci. 54, 529–550. 10.1080/10408363.2017.1410520 29226748 PMC6733278

[B29] Gerlinger M. Swanton C. (2010). How Darwinian models inform therapeutic failure initiated by clonal heterogeneity in cancer medicine. Br. J. Cancer 103, 1139–1143. 10.1038/sj.bjc.6605912 20877357 PMC2967073

[B30] Gerson S. (2004). MGMT: its role in cancer aetiology and cancer therapeutics. Nat. Rev. Cancer. 4, 296–307. 10.1038/nrc1319 15057289

[B31] Ginsburg G. S. Phillips K. A. (2018). Precision medicine: from science to value. Health Aff. (Millwood). 37, 694–701. 10.1377/hlthaff.2017.1624 29733705 PMC5989714

[B32] Gracia A. Balañá C. Kaskens L. Chiavenna S. Matias-Guiu X. Rubio-Rodríguez D. (2015). Economic analysis of epicup, an epigenetic test to predict the tissue of origin in cancer of unknown primary site, the USA payors perspective. Value Heal. 18, A356. 10.1016/j.jval.2015.09.670

[B33] Gradíssimo A. Burk R. D. (2017). Molecular tests potentially improving HPV screening and genotyping for cervical cancer prevention. Expert Rev. Mol. Diagn. 17, 379–391. 10.1080/14737159.2017.1293525 28277144 PMC5904788

[B34] Gusyatiner O. Hegi M. E. (2018). Glioma epigenetics: from subclassification to novel treatment options. Semin. Cancer Biol. 51, 50–58. 10.1016/j.semcancer.2017.11.010 29170066

[B35] Harbeck N. Gnant M. (2017). Breast cancer. Lancet 389, 1134–1150. 10.1016/S0140-6736(16)31891-8 27865536

[B36] Hartmann O. Spyratos F. Harbeck N. Dietrich D. Fassbender A. Schmitt M. (2009). DNA methylation markers predict outcome in node-positive, estrogen receptor-positive breast cancer with adjuvant anthracycline-based chemotherapy. Clin. Cancer Res. 15, 315–323. 10.1158/1078-0432.CCR-08-0166 19118060

[B37] Hashimoto Y. Zumwalt T. J. Goel A. (2016). DNA methylation patterns as noninvasive biomarkers and targets of epigenetic therapies in colorectal cancer. Epigenomics 8, 685–703. 10.2217/epi-2015-0013 27102979 PMC4928499

[B38] He N. Song L. Kang Q. Jin P. Cai G. Zhou J. (2018). The pathological features of colorectal cancer determine the detection performance on blood ctDNA. Technol. Cancer Res. Treat. 17, 153303381879179. 10.1177/1533033818791794 PMC6144579 30223720

[B39] Holdenrieder S. Dharuman Y. Standop J. Trimpop N. Herzog M. Hettwer K. (2014). Novel serum nucleosomics biomarkers for the detection of colorectal cancer. Anticancer Res. 34 (5), 2357–2362. 24778043

[B40] Holder L. B. Haque M. M. Skinner M. K. (2017). Machine learning for epigenetics and future medical applications. Epigenetics 12, 505–514. 10.1080/15592294.2017.1329068 28524769 PMC5687335

[B41] Hsu C.-Y. Ho H.-L. Lin S.-C. Chen M.-H. Hsu S. P.-C. Yen Y.-S. (2017). Comparative assessment of 4 methods to analyze MGMT status in a series of 121 glioblastoma patients. Appl. Immunohistochem. Mol. Morphol. AIMM 25, 497–504. 10.1097/PAI.0000000000000331 27153440

[B42] Ilse P. Biesterfeld S. Pomjanski N. Fink C. Schramm M. (2013). SHOX2 DNA methylation is a tumour marker in pleural effusions. Cancer Genomics Proteomics 10 (5), 217–223. 24136974

[B43] Imperiale T. F. Ransohoff D. F. Itzkowitz S. H. Levin T. R. Lavin P. Lidgard G. P. (2014). Multitarget stool DNA testing for colorectal-cancer screening. N. Engl. J. Med. 370, 1287–1297. 10.1056/NEJMoa1311194 24645800

[B44] Issa I. A. Noureddine M. (2017). Colorectal cancer screening: an updated review of the available options. World J. Gastroenterol. 23, 5086–5096. 10.3748/wjg.v23.i28.5086 28811705 PMC5537177

[B45] Jin P. Kang Q. Wang X. Yang L. Yu Y. Li N. (2015). Performance of a second-generation methylated SEPT9 test in detecting colorectal neoplasm. J. Gastroenterol. Hepatol. 30, 830–833. 10.1111/jgh.12855 25471329

[B46] Johannessen L. Bradal P. Myklebust T. A. Heim S. Micci F. Panagopoulos I. (2018). MGMT gene promoter methylation status – assessment of two pyrosequencing kits and three methylation-specific PCR methods for their predictive capacity in glioblastomas. Cancer Genomics Proteomics 15, 437–446. 10.21873/cgp.20102 30343277 PMC6299788

[B47] Kern S. E. (2012). Why your new cancer biomarker may never work: recurrent patterns and remarkable diversity in biomarker failures. Cancer Res. 72, 6097–6101. 10.1158/0008-5472.CAN-12-3232 23172309 PMC3513583

[B48] Kneip C. Schmidt B. Seegebarth A. Weickmann S. Fleischhacker M. Liebenberg V. (2011). SHOX2 DNA methylation is a biomarker for the diagnosis of lung cancer in plasma. J. Thorac. Oncol. 6, 1632–1638. 10.1097/JTO.0b013e318220ef9a 21694641

[B49] Kumar V. Abbas A. K. Fausto N. Mitchell R. (2007). Robbins Basic Pathology (8th ed). 8th ed Philadelphia: Saunders Elseviers.

[B50] Lai H. Lin Y. Huang R. Chung M. Wang H. Liao Y. (2008). Identification of novel DNA methylation markers in cervical cancer. Int. J. Cancer. 123 (1), 161–167. 10.1002/ijc.23519 18398837

[B51] Lai H. Lin Y. Huang R. Chung M. Wang H. Liao Y. (2010). Quantitative DNA methylation analysis detects cervical intraepithelial neoplasms type 3 and worse. Cancer. 116 (8), 4266–4274. 10.1002/cncr.25252 20564139

[B52] Lantuéjoul S. Salameire D. Salon C. Brambilla E. (2009). Pulmonary preneoplasia–sequential molecular carcinogenetic events. Histopathology 54, 43–54. 10.1111/j.1365-2559.2008.03182.x 19187179

[B53] Lauby-Secretan B. Scoccianti C. Loomis D. Benbrahim-Tallaa L. Bouvard V. Bianchini F. (2015). Breast-cancer screening—viewpoint of the IARC Working Group. N. Engl. J. Med. 372, 2353–2358. 10.1056/NEJMsr1504363 26039523

[B54] Laurent-Puig P. Grisoni M.-L. Heinemann V. Bonnetain F. Fontaine K. Vazart C. (2017). MiR 31 3p as a predictive biomarker of cetuximab efficacy effect in metastatic colorectal cancer (mCRC) patients enrolled in FIRE-3 study. J. Clin. Oncol., 34, 3516–3516. 10.1200/JCO.2016.34.15_suppl.3516

[B55] Laurent-Puig P. Grisoni M.-L. Heinemann V. Liebaert F. Neureiter D. Jung A. (2019). Validation of miR-31-3p expression to predict cetuximab efficacy when used as first-line treatment in RAS wild-type metastatic colorectal cancer. Clin. Cancer Res. 25, 134–141. 10.1158/1078-0432.CCR-18-1324 30108104

[B56] Laurent-Puig P. Paget-Bailly S. Vernerey D. Vazart C. Decaulne V. Fontaine K. (2015). Evaluation of miR 31 3p as a biomarker of prognosis and panitumumab benefit in RAS -wt advanced colorectal cancer (aCRC): analysis of patients (pts) from the PICCOLO trial. J. Clin. Oncol. 33, 3547–3547. 10.1200/jco.2015.33.15_suppl.3547

[B57] Lewin J. Plum A. Hildmann T. Rujan T. Eckhardt F. Liebenberg V. (2007). Comparative DNA methylation analysis in normal and tumour tissues and in cancer cell lines using differential methylation hybridisation. Int. J. Biochem. Cell Biol. 39, 1539–1550. 10.1016/j.biocel.2007.03.006 17499000

[B58] Li J. Lenferink A. E. G. Deng Y. Collins C. Cui Q. Purisima E. O. (2010). Identification of high-quality cancer prognostic markers and metastasis network modules. Nat. Commun. 1, 1–8. 10.1038/ncomms1033 20975711 PMC2972666

[B59] Li N. He Y. Mi P. Hu Y. (2019). ZNF582 methylation as a potential biomarker to predict cervical intraepithelial neoplasia type III/worse: a meta-analysis of related studies in Chinese population. Medicine (Baltimore) 98, e14297. 10.1097/MD.0000000000014297 30732145 PMC6380660

[B60] Lidgard G. Domanico M. Bruinsma J. Light J. Gagrat Z. Oldham-Haltom R. (2013). Clinical performance of an automated stool DNA assay for detection of colorectal neoplasia. Clin. Gastroenterol. Hepatol. 11, 1313–1318. 10.1016/j.cgh.2013.04.023 23639600

[B61] Lin H. Chen T.-C. Chang T.-C. Cheng Y.-M. Chen C.-H. Chu T.-Y. (2014). Methylated ZNF582 gene as a marker for triage of women with Pap smear reporting low-grade squamous intraepithelial lesions—a Taiwanese Gynecologic Oncology Group (TGOG) study. Gynecol. Oncol. 135, 64–68. 10.1016/j.ygyno.2014.08.012 25134998

[B62] Lin J. Piper M. Perdue L. Rutter C. Webber E. O’Connor E. (2016). Screening for colorectal cancer: updated evidence report and systematic review for the US Preventive Services Task Force. JAMA 315, 2576–2594. 10.1001/jama.2016.3332 27305422 PMC13623242

[B63] Liou Y.-L. Zhang T.-L. Yan T. Yeh C.-T. Kang Y.-N. Cao L. (2016). Combined clinical and genetic testing algorithm for cervical cancer diagnosis. Clin. Epigenetics 8, 66. 10.1186/s13148-016-0232-3 27293491 PMC4902988

[B64] Lombardi M. Y. Assem M., (2017). Glioblastoma Genomics: A Very Complicated Story. ed. De Vleeschouwer S., Brisbane, AU: Codon Publications, p. 3–25. 10.15586/codon.glioblastoma.2017.ch1 29251861

[B65] Louis D. N. Perry A. Reifenberger G. von Deimling A. Figarella-Branger D. Cavenee W. K. (2016). The 2016 World Health Organization classification of tumors of the central nervous system: a summary. Acta Neuropathol. 131, 803–820. 10.1007/s00401-016-1545-1 27157931

[B66] Low S.-K. Zembutsu H. Nakamura Y. (2018). Breast cancer: the translation of big genomic data to cancer precision medicine. Cancer Sci. 109, 497–506. 10.1111/cas.13463 29215763 PMC5834810

[B67] Maier S. Nimmrich I. Koenig T. Eppenberger-Castori S. Bohlmann I. Paradiso A. (2007). DNA-methylation of the homeodomain transcription factor PITX2 reliably predicts risk of distant disease recurrence in tamoxifen-treated, node-negative breast cancer patients—technical and clinical validation in a multi-centre setting in collaboration wi. Eur. J. Cancer 43, 1679–1686. 10.1016/j.ejca.2007.04.025 17601725

[B68] Marielle H. Mark E. Jake M. Dorian P. Brieuc C. Jason T. (2017). Validation of Nu.QTM colorectal cancer screening triage test to identify FIT positive individuals at low risk of screen relevant neoplasia. Ann. Oncol. 28 (3), mdx262.021. 10.1093/annonc/mdx262.021

[B69] Meisel J. L. Venur V. A. Gnant M. Carey L. (2018). Evolution of targeted therapy in breast cancer: where precision medicine began. Am. Soc. Clin. Oncol. Educ. Book, 38, 78–86. 10.1200/EDBK_201037 30231395

[B70] Mitchell S. M. Ho T. Brown G. S. Baker R. T. Thomas M. L. McEvoy A. (2016). Evaluation of methylation biomarkers for detection of circulating tumor DNA and application to colorectal cancer. Genes (Basel). 7, 125, 10.3390/genes7120125 PMC5192501 27983717

[B71] Moran S. Martínez-Cardús A. Sayols S. Musulén E. Balañá C. Estival-Gonzalez A. (2016). Epigenetic profiling to classify cancer of unknown primary: a multicentre, retrospective analysis. Lancet Oncol. 17, 1386–1395. 10.1016/S1470-2045(16)30297-2 27575023

[B72] Morikawa T. Kato J. Yamaji Y. Wada R. Mitsushima T. Shiratori Y. (2005). A comparison of the immunochemical fecal occult blood test and total colonoscopy in the asymptomatic population. Gastroenterology 129, 422–428. 10.1016/j.gastro.2005.05.056 16083699

[B73] Niu F. Wen J. Fu X. Li C. Zhao R. Wu S. (2017). Stool DNA test of methylated syndecan-2 for the early detection of colorectal neoplasia. Cancer Epidemiol. Biomarkers Prev. 26, 1411–1419. 10.1158/1055-9965.EPI-17-0153 28619831

[B74] Oh T. J. Oh H. Seo Y. Y. Jeong D. Kim C. Kang H. W. (2017). Feasibility of quantifying SDC2 methylation in stool DNA for early detection of colorectal cancer. Clin. Epigenetics 9, 126. 10.1186/s13148-017-0426-3 29225717 PMC5715626

[B75] Oh T. Kim N. Moon Y. Kim M. S. Hoehn B. D. Park C. H. (2013). Genome-wide identification and validation of a novel methylation biomarker, SDC2, for blood-based detection of colorectal cancer. J. Mol. Diagnostics 15, 498–507. 10.1016/j.jmoldx.2013.03.004 23747112

[B76] Ørntoft M. Nielsen H. Ørntoft T. Andersen C. (2015). Performance of the colorectal cancer screening marker Sept9 is influenced by age, diabetes and arthritis: a nested case-control study. BMC Cancer 819. 10.1186/s12885-015-1832-6 26514170 PMC4625973

[B77] Panagopoulos I. Gorunova L. Leske H. Niehusmann P. Johannessen L. E. Staurseth J. (2018). Pyrosequencing analysis of MGMT promoter methylation in meningioma. Cancer Genomics Proteomics 15, 379–385. 10.21873/cgp.20096 30194078 PMC6199576

[B78] Park Y. S. Kim D. S. Cho S. W. Park J. W. Jeon S. J. Moon T. J. (2018). Analysis of syndecan-2 methylation in bowel lavage fluid for the detection of colorectal neoplasm. Gut Liver 12, 508–515. 10.5009/gnl17357 29730903 PMC6143447

[B79] Pavlidis N. Fizazi K. (2009). Carcinoma of unknown primary (CUP). Crit. Rev. Oncol. Hematol. 69, 271–278. 10.1016/j.critrevonc.2008.09.005 18977667

[B80] Pavlidis N. Pentheroudakis G. (2012). Cancer of unknown primary site. Lancet 379, 1428–1435. 10.1016/S0140-6736(11)61178-1 22414598

[B81] Pentheroudakis G. Pavlidis N. Fountzilas G. Krikelis D. Goussia A. Stoyianni A. (2013). Novel microRNA-based assay demonstrates 92% agreement with diagnosis based on clinicopathologic and management data in a cohort of patients with carcinoma of unknown primary. Mol. Cancer 12, 57. 10.1186/1476-4598-12-57 23758919 PMC3695805

[B82] Perkins J. Napieralski R. Schricker G. Piednoir E. Manner O. Bona A. (2018). TherascreenPITX2 RGQ PCR assay for the assessment of PITX2 DNA-methylation status to investigate the role of the transcription factor PITX2 and the regulation of the Wnt/ß-catenin pathway in pathophysiological processes. Protoc. Exch. 10.1038/protex.2018.022

[B83] Potter N. Hurban P. White M. Whitlock K. Lofton-Day C. Tetzner R. (2014). Validation of a real-time PCR-based qualitative assay for the detection of methylated SEPT9 DNA in human plasma. Clin. Chem. 60, 1183–1191. 10.1373/clinchem.2013.221044 24938752

[B84] Pugh S. Thiébaut R. Bridgewater J. Grisoni M.-L. Moutasim K. Rousseau F. (2017). Association between miR-31-3p expression and cetuximab efficacy in patients with KRAS wild-type metastatic colorectal cancer: a post-hoc analysis of the New EPOC trial. Oncotarget. 8, 93856–93866. 10.18632/oncotarget.21291 29212194 PMC5706840

[B85] Quillien V. Lavenu A. Ducray F. Meyronet D. Chinot O. Fina F. (2017). Clinical validation of the CE-IVD marked Therascreen MGMT kit in a cohort of glioblastoma patients. Cancer Biomarkers 20, 435–441. 10.3233/CBM-170191 28800313

[B86] Quillien V. Lavenu A. Karayan-Tapon L. Carpentier C. Labussiãre M. Lesimple T. (2012). Comparative assessment of 5 methods (methylation-specific polymerase chain reaction, methylight, pyrosequencing, methylation-sensitive high-resolution melting, and immunohistochemistry) to analyze O6-methylguanine-DNA-methyltranferase in a series of 100. Cancer 118, 4201–4211. 10.1002/cncr.27392 22294349

[B87] Rahier J. Druez A. Faugeras L. Martinet J. Géhénot M. Josseaux E. (2017). Circulating nucleosomes as new blood-based biomarkers for detection of colorectal cancer. Clin. Epigenetics. 9, 53. 10.1186/s13148-017-0351-5 28515797 PMC5433015

[B88] Ramon L. David C. Fontaine K. Lallet E. Marcaillou C. Martin-Lannerée S. (2018). Technical validation of a reverse-transcription quantitative polymerase chain reaction in vitro diagnostic test for the determination of MiR-31-3p expression levels in formalin-fixed paraffin-embedded metastatic colorectal cancer tumor specimens. Biomark. Insights 13, 1177271918763357. 10.1177/1177271918763357 29568219 PMC5858679

[B89] Schmidt B. Liebenberg V. Dietrich D. Schlegel T. Kneip C. Seegebarth A. (2010). SHOX2 DNA methylation is a biomarker for the diagnosis of lung cancer based on bronchial aspirates. BMC Cancer 10, 600. 10.1186/1471-2407-10-600 21047392 PMC2988753

[B90] Schricker G. Napieralski R. Noske A. Piednoir E. Manner O. Schüren E. (2018). Clinical performance of an analytically validated assay in comparison to microarray technology to assess PITX2 DNA-methylation in breast cancer. Sci. Rep. 8, 16861. 10.1038/s41598-018-34919-1 30442983 PMC6237923

[B91] Shen-Gunther J. Wang C.-M. Poage G. M. Lin C.-L. Perez L. Banks N. A. (2016). Molecular Pap smear: HPV genotype and DNA methylation of ADCY8, CDH8, and ZNF582 as an integrated biomarker for high-grade cervical cytology. Clin. Epigenetics 8, 96. 10.1186/s13148-016-0263-9 27651839 PMC5022163

[B92] Shen C. Changxin Z. Wei L. Shanping S. Anqi Z. Wenqiang T. (2018). Methylated septin 9 gene for noninvasive diagnosis and therapy monitoring of breast cancer. Transl. Cancer Res. 7, 587–599. 10.21037/tcr.2018.05.24

[B93] Sheng X. Guo Y. Lu Y. (2017). Prognostic role of methylated GSTP1, p16, ESR1 and PITX2 in patients with breast cancer. Medicine (Baltimore) 96, e7476. 10.1097/MD.0000000000007476 28700487 PMC5515759

[B94] Siegel R. L. Miller K. D. Jemal A. (2017). Cancer statistics, 2017. CA. Cancer J. Clin. 67, 7–30. 10.3322/caac.21387 28055103

[B95] Siegel R. Naishadham D. Jemal A. (2012). Cancer statistics, 2012. CA. Cancer J. Clin. 62, 10–29. 10.3322/caac.20138 22237781

[B96] Song L. Li Y. (2015). Methylated Sept9 gene is a sensitive biomarker for all stages of colorectal cancer. iMedPub J. 1 (1), 1–7. 10.21767/2471-9943.100002

[B97] Song L. Wang J. Wang H. Chen Y. Jia J. Guo S. (2018). The quantitative profiling of blood mSEPT9 determines the detection performance on colorectal tumors. Epigenomics 10, 1569–1583. 10.2217/epi-2017-0154 30426784

[B98] Taieb J. Tabernero J. Mini E. Subtil F. Folprecht G. Van Laethem J. (2014). Oxaliplatin, fluorouracil, and leucovorin with or without cetuximab in patients with resected stage III colon cancer (PETACC-8): an open-label, randomised phase 3 trial. Lancet Oncol., 15 (8), 862–873. 10.1016/S1470-2045(14)70227-X 24928083

[B99] Torre L. A. Islami F. Siegel R. L. Ward E. M. Jemal A. (2017). Global cancer in women: burden and trends. Cancer Epidemiol. Biomarkers Prev. 26, 444–457. 10.1158/1055-9965.EPI-16-0858 28223433

[B100] Torre L. A. Siegel R. L. Ward E. M. Jemal A. (2016). Global cancer incidence and mortality rates and trends—an update. Cancer Epidemiol. Biomarkers Prev. 25, 16–27. 10.1158/1055-9965.EPI-15-0578 26667886

[B101] van Lanschot M. C. J. Carvalho B. Coupé V. M. H. van Engeland M. Dekker E. Meijer G. A. (2017). Molecular stool testing as an alternative for surveillance colonoscopy: a cross-sectional cohort study. BMC Cancer 17, 116. 10.1186/s12885-017-3078-y 28173852 PMC5297018

[B102] Varadhachary G. R. Raber M. N. (2014). Cancer of unknown primary site. N. Engl. J. Med. 371, 757–765. 10.1056/NEJMra1303917 25140961

[B103] Vu M. Yu J. Awolude O. A. Chuang L. (2018). Cervical cancer worldwide. Curr. Probl. Cancer 42, 457–465. 10.1016/j.currproblcancer.2018.06.003 30064936

[B104] Weiss G. Schlegel A. Kottwitz D. König T. Tetzner R. (2017). Validation of the SHOX2/PTGER4 DNA Methylation marker panel for plasma-based discrimination between patients with malignant and nonmalignant lung disease. J. Thorac. Oncol. 12, 77–84. 10.1016/j.jtho.2016.08.123 27544059 PMC5226366

[B105] Wentzensen N. Sherman M. E. Schiffman M. Wang S. S. (2009). Utility of methylation markers in cervical cancer early detection: appraisal of the state-of-the-science. Gynecol. Oncol. 112, 293–299. 10.1016/j.ygyno.2008.10.012 19054549 PMC2673716

[B106] Widschwendter M. Siegmund K. D. Müller H. M. Fiegl H. Marth C. Müller-Holzner E. (2004). Association of breast cancer DNA methylation profiles with hormone receptor status and response to tamoxifen. Cancer Res. 64, 3807–3813. 10.1158/0008-5472.CAN-03-3852 15172987

[B107] Zawlik I. Vaccarella S. Kita D. Mittelbronn M. Franceschi S. Ohgaki H. (2009). Promoter methylation and polymorphisms of the MGMT gene in glioblastomas: a population-based study. Neuroepidemiology 32, 21–29. 10.1159/000170088 18997474

[B108] Zhao H. Wang S. Song C. Zha Y. Li L. (2016). The prognostic value of MGMT promoter status by pyrosequencing assay for glioblastoma patients’ survival: a meta-analysis. World J. Surg. Oncol. 14, 261. 10.1186/s12957-016-1012-4 27733166 PMC5062843

[B109] Zou H. Allawi H. Cao X. Domanico M. Harrington J. Taylor W. (2012). Quantification of methylated markers with a multiplex methylation-specific technology. Clin. Chem. 58, 375–383. 10.1373/clinchem.2011.171264 22194633

